# Health related quality of life after oesophagectomy: elderly patients refer similar eating and swallowing difficulties than younger patients

**DOI:** 10.1186/s12885-015-1647-5

**Published:** 2015-09-21

**Authors:** Francesco Cavallin, Eleonora Pinto, Luca M. Saadeh, Rita Alfieri, Matteo Cagol, Carlo Castoro, Marco Scarpa

**Affiliations:** Surgical Oncology Unit, Regional Centre for Oesophageal Disease, Veneto Institute of Oncology IOV IRCCS, Via Gattamelata 64, 35128 Padua, Italy

**Keywords:** Elderly, Quality of life, Oesophagectomy

## Abstract

**Background:**

Oesophagectomy for cancer could be safe and worthwhile in selected older patients, but less is known about the effect of oesophagectomy on perceived quality of life of such delicate class of cancer patients. The aim of this study was to evaluate the impact of oesophagectomy for cancer in elderly patients in term of health-related quality of life.

**Methods:**

We retrospectively evaluated all consecutive patients who underwent oesophagectomy for cancer at the Surgical Oncology Unit of the Veneto Institute of Oncology between November 2009 and March 2014. Quality of life was evaluated using EORTC C-30 and OES-18 questionnaires at admission, at discharge and 3 months after surgery. Adjusted multivariable linear mixed effect models were estimated to assess mean score differences (MDs) of selected aspects in older (≥70 years) and younger (<70 years) patients.

**Results:**

Among 109 participating patients, 23 (21.1 %) were at least 70 years old and 86 (78.9 %) were younger than 70 years. Global quality of life was clinically similar between older and younger patients over time (MD 4.4). Older patients reported clinically and statistically significantly worse swallowing saliva (MD 17.4, 95 % C.I. 3.6 to 31.2), choking when swallowing (MD 13.8, 95 % C.I. 5.8 to 21.8) and eating difficulties (MD 20.1 95 % C.I. 7.4 to 32.8) than younger patients only at admission.

**Conclusions:**

Early health-related quality of life perception after surgery resulted comparable in older and younger patients. This result may also be due to some predisposition of the elderly to adapt to the new status.

**Electronic supplementary material:**

The online version of this article (doi:10.1186/s12885-015-1647-5) contains supplementary material, which is available to authorized users.

## Background

The increment of life expectancy is leading to an increasing number of older individuals, requiring care for age-related disorders. Health policies are addressing this situation by making more efforts to manage and sustain elderly care system [1]. The management of elderly is even more delicate in cancer patients because such patients have unique issues that require evaluation of life expectancy, functional status, comorbidities and risk of treatment-related morbidity [2].

**Fig. 1:**
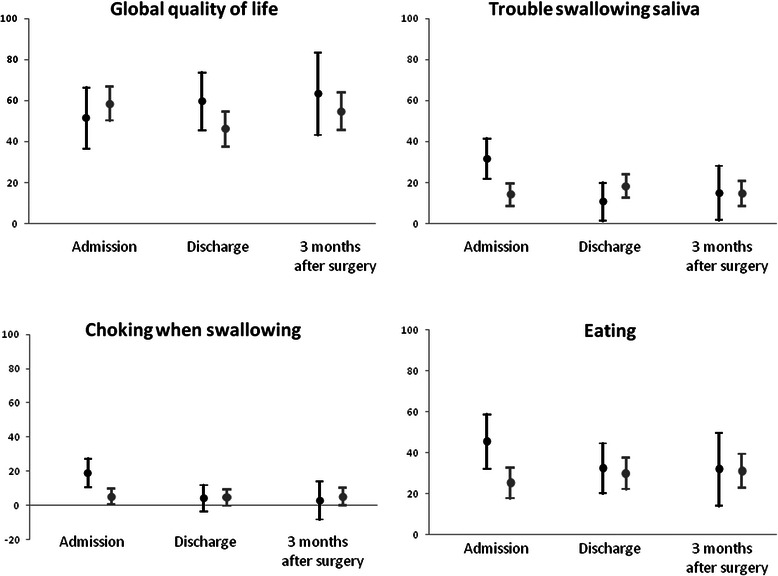
HRQL in older (*black*) and younger (*grey*) patients at admission, at discharge and at 3 months after surgery. Legend: Estimates of selected EORTC aspects (with 95 % Confidence Intervals) as calculated by linear mixed-effect models, adjusting for time, Charlson Comorbidity Index, neoadjuvant therapy, tumour stage, gender, histology, tumour site and complications.. A significant group-by-time interaction (*p* < 0.01) in OES18 trouble swallowing saliva, OES18 choking when swallowing and OES18 eating suggested more problems in older patients than younger ones at admission, but not at discharge and at 3 months after surgery. C30 global quality of life did not change over time (*p* = 0.57)

Oesophageal cancer is the eighth most common incident cancer in the world and the incidence is rapidly increasing. The median age of oesophageal cancer patients is around 65-68 years, therefore the ageing of the population and the longer life expectancy have led to more elderly patients with oesophageal cancers being referred for treatment [3, 4]. Although the standard curative treatment includes oesophagectomy [5], elderly patients with oesophageal cancer are less likely to undergo surgery because their age-related comorbidities can affect the outcome [4]. However, several studies demonstrated that oesophagectomy could be safe and worthwhile in selected elderly patients who are fit for surgery and have resectable lesions [6–9].

In addition to morbidity and mortality, the health-related quality of life (HRQL) has become an important outcome measure of surgical treatment for cancer [10] and recent systematic reviews showed a deterioration in HRQL after oesophagectomy [11–13]. Due to frailty and medical comorbidities, elderly patients could be expected to experience a worse impairment of HRQL after surgical treatment, even if they are selected as surgical candidates. Therefore, the aim of this retrospective study was to evaluate the impact of oesophagectomy for cancer in elder patients in term of HRQL.

## Methods

### Study design

All consecutive patients who underwent oesophagectomy for cancer at the Surgical Oncology Unit of the Veneto Institute of Oncology between November 2009 and March 2014 were retrospectively included in the present study using a prospectively collected database. Since November 2009, all patients selected for surgery at out Unit were invited to fill questionnaires about quality of life at different time points [14]. Health related quality of life (HRQL) was assessed using the EORTC QLQ-C30 and QLQ-OES18 questionnaires that were collected at admission for surgery, at discharge and 3 months after discharge. The patients manually filled the questionnaires in the ward (first and second time points) and in the outpatient clinic (third time point). Validated translation in Italian language was used. Patients who answered the questionnaires were included in the final analysis on quality of life and they were divided in two groups (<70 vs. ≥70 years old), according to previous studies [15–17]. Patients who did not answer the questionnaires were compared to those who answered in term of demographic and tumour characteristics. The study was conducted according to Helsinki Declaration principles and all patients (who completed or not the questionnaire) gave their written consent to have their data collected for scientific purpose. This retrospective study was notified to the Reasearch Ethics Committee of the Veneto Institute of Oncology (protocol number 0014057; protocol code AEC). According to the Italian Law, observational retrospective studies (not involving any drugs) do not need approval from local IRB. Anyway, we usually notify our studies to our IRB (the Research Ethics Committee of the Veneto Institute of Oncology) according to internal policy, in order to assess the absence of any ethical problems. The Research Ethics Committee of the Veneto Institute of Oncology examined our study and communicated the absence of any ethical problems concerning the procedures of the research or patients’ rights.

### Health-related quality of life

The EORTC QLQ-C30 is a 30-items questionnaire for assessing the generic quality of life of cancer patients [18] and it is widely used by cancer research groups. The QLQ-OES18 is the specific module for oesophageal cancer and it is designed for patients with local, locally advanced or metastatic disease treated with single or combination treatment including surgery, chemotherapy, radiotherapy or endoscopic palliation [19]. Specific aspects were selected *a priori* as outcome of the study, whereas the remaining aspects were not analyzed. The selected aspects were C30 global quality of life, all C30 functional scales (physical function, role function, social function, emotional function, cognitive function), C30 fatigue, C30 dyspnoea, OES18 dysphagia, OES18 trouble swallowing saliva, OES18 choking when swallowing and OES18 eating. All scores range from 0 to 100, with a high score representing healthy status for functional scales and the global health status scale, but high level of symptomatology/problems for symptom scales (apart from OES18 dysphagia).

### Pre-operative evaluation

Tumour staging was performed according to the criteria of the International Union Against Cancer [20]. Preoperative staging was performed in all patients including: esophagoscopy with endoscopic ultrasound, high resolution computed tomography scan of the thorax and abdomen, and integrated fluorodeoxyglucose positron emission tomography/computed tomography. For carcinomas of the upper intrathoracic oesophagus, bronchoscopy and cervical ultrasound were performed. Patients’ comorbidities were evaluated using the Charlson Comorbidity Index (CCI) [21] and the age related Charlson Comorbidity Index (ACCI) [22]. Nutritional status of the patients was assessed using the prognostic nutritional index (PNI), which includes albumin level and total lymphocyte count [23].

### Indication for treatment

Indication for surgery was evaluated by an experienced multidisciplinary team composed of a dedicated upper GI surgeon, a medical oncologist and radiation oncologist. Patients with tumour staged above T3N0 or anyTN1 were offered neoadjuvant therapy as described elsewhere [14]. Patients were considered resectable when staged below T3N0 or, after the termination of neoadjuvant treatment, when there was no evidence of distant metastases or locally advanced tumour with gross periesophageal involvement at restaging.

### Evaluation of fitness for surgery

All patients underwent detailed risk assessment based on history of chronic pulmonary disease, cardiovascular, hepatic and renal disease, which included: routine biochemical profile, electrocardiography, echocardiography and cardiac stress test (when indicated), pulmonary function tests with spirometry and arterial gas analysis. Fitness for surgery was assessed by an experienced multisciplinary team composed of a dedicated upper GI surgeon, an anaesthesiologist, a cardiologist and a pulmonologist. Patients were considered unfit for surgery when severe respiratory insufficiency, cardiac failure, severe renal or hepatic disease was found. If possible, patients were referred for specific treatment of the underlying disease and then re-evaluated. If this was not the case and the perioperative risk was judged to be too high, the multidisciplinary team referred the patients for definitive chemo-radiotherapy or palliation of symptoms.

### Surgical treatment

Details concerning surgical techniques have been published elsewhere [8]. Briefly, oesophagectomy was performed using an Ivor-Lewis procedure, via a laparotomy and right thoracotomy, for tumours of the mid-lower oesophagus and gastric cardia. A three-stage McKeown’s procedure, with an additional left cervical incision, was reserved for tumours in the upper third of the oesophagus. At least 6–8 cm of healthy oesophagus was resected above the proximal edge of the tumour to avoid neoplastic involvement of the resection margins (in case of cervical oesophageal cancer, the portion of resected healthy oesophagus was smaller). In this group of patients en bloc lymph node dissection was performed, including the paraesophageal, sub carinal, posterior mediastinal and paracardial lymph nodes, as well as those located along the lesser gastric curvature, the origin of the left gastric artery, the celiac trunk, the common hepatic artery and the splenic artery. The alimentary tract was reconstructed using the gastric pull-up technique; if the stomach was unavailable, either a jejunal loop or the left colon was used [24].

### Statistical analysis

Continuous data were expressed as median and interquartile range (IQR). Categorical data were compared between two groups using Fisher’s test and continuous data using Mann–Whitney test.

Linear mixed effect models were used to assess mean score differences (MDs) with 95 % confidence intervals (95 % C.I.) for the selected HRQL aspects, accounting for the longitudinal structure of the data [25]. Missing data were handled according to EORTC developers’ recommendations [18, 19]. The main cause of missing data was staff oversights, thus the assumption of “missing at random” can be justified in our series. Comparisons were performed between older and younger patients adjusting for a set of potential confounders (time, Charlson Comorbidity Index, neoadjuvant therapy, tumour stage, gender, histology, tumour site and complications). A group-by-time interaction factor was included in the models and tested for statistical significance. A significant interaction factor meant that the MD between older and younger patients varied over time and in such case the two age groups were compared at each time point. A non-significant interaction factor meant that the MD between older and younger patients persisted over time, thus the interaction term was removed from the model. In such case, the model estimated a single MD for the whole period, because the removal of the interaction factor implied a parallel variation of the two age groups over time. Any MD ≥ 10 points was considered clinically significant according to developers [18, 26] and tested for statistical significance. Any MD < 10 points was considered clinically not significant thus was not tested for statistical significance and was indicated as “n.a.” (not applicable) [25]. This approach contributed to avoid the risk of multiple testing. Statistical analysis was performed using SAS 9.1 and a p-value less than 0.05 was considered significant.

## Results

### Sample characteristics

One hundred and eighty-three patients underwent oesophagectomy for cancer at the Surgical Oncology Unit of the Veneto Institute of Oncology between November 2009 and March 2014. Seventy-four of them (40.4 %) refused to fill the questionnaires. They were similar to participating patients about demographic and tumour characteristics, but they were less likely to receive neoadjuvant therapy (68.9 % vs. 82.6 %, *p* = 0.05) and had shorter hospital stays (median 14 days vs. 19 days, *p* < 0.0001; Table 1).

**Table 1:** Comparison between patients who did and did not fill the questionnaires

	Patients who did not fill the questionnaires	Patients who filled the questionnaires	*p*-value
N	74	109	-
Gender Male: Female	60:14	92:17	0.55
Age (years)^a^	63 (54–70)	61 (53–68)	0.35
Histotype:^b^			0.75
Adenocarcinoma	49 (66.2)	75 (69.4)	
Squamous cell carcinoma	25 (33.8)	33 (30.6)	
Tumour site:			0.08
Cervical oesophagus	4 (5.4)	6 (5.5)	
Thoracic oesophagus	19 (26.7)	14 (12.8)	
Oesophago-gastric junction	51 (68.9)	89 (81.7)	
Neoadjuvant therapy: yes	51 (68.9)	90 (82.6)	0.05
Hospital stay (days)^a^	14 (13–20)	19 (17–24)	<0.0001
Pathological stage^c^			0.16
0	8 (10.8)	25 (23.0)	
I	18 (24.3)	19 (17.4)	
II	18 (24.3)	24 (22.0)	
III	29 (39.2)	36 (33.0)	
IV	1 (1.4)	5 (4.6)	
Post-operative morbidity	22 (29.7)	29 (26.6)	0.74
Type of post-operative complications:			
Anastomotic leak	6	8	
Pulmonary	4	17	
Cardiac	7	9	
Dysphonia	0	3	
Urinary	3	4	
Bleeding	3	1	
Sepsis	0	1	
Chylothorax	1	0	
Diaframmatic hernia	0	1	
Deep venous thrombosis	0	1	
Pyloric stenosis	1	0	-

Data expressed as n (%) or ^a^median (IQR)

^b^Other histotype in one patient

^c^Pathological stage is ypstage for patients who received neoadjuvant therapy and pstage for patients who did not

### Patients who filled the questionnaires

The characteristics of the 109 patients who underwent oesophagectomy and filled the questionnaire are shown in Table 2. Twenty-three (21.1 %) were at least 70 years old and 86 (78.9 %) were younger than 70 years. Older patients were less likely to receive neoadjuvant therapy (65.2 % vs. 87.2 %, *p* = 0.03) and have longer hospital stay than younger patients (median 23 days vs. 19 days, *p* = 0.04). Two elderly patients underwent surgery without neoadjuvant therapy due to cardiac comorbidity in conjunction with advanced age. In addition, older patients tended to have higher comorbidity status than younger patients (CCI *p* = 0.09 and ACCI *p* < 0.0001, due to age penalization in score calculation; Table 2).

**Table 2:** Patients who filled the questionnaires: younger vs. older patients

	Age < 70	Age > =70	*p*-value
N	86	23	-
Gender Male: Female	73:13	19:4	0.75
Age (years)^a^	58 (51–64)	75 (72–80)	<0.0001
Histotype:^b^			0.14
Adenocarcinoma	62 (72.9)	13 (56.5)	
Squamous cell carcinoma	23 (27.1)	10 (43.5)	
Tumour site:			0.42
Cervical oesophagus	6 (7.0)	0	
Thoracic oesophagus	10 (11.6)	4 (17.4)	
Oesophago-gastric junction	70 (81.4)	19 (82.6)	
Prognostic nutritional index^a^	49.0 (46.5–52.0)	48.2 (44.4–50.7)	0.39
Charlson Comorbidity Index^a^	2 (2–3)	3 (2–3)	0.09
Age related Charlson Comorbidity Index^ac^	3 (2–4)	5 (5–6)	<0.0001
Neoadjuvant therapy	75 (87.2)	15 (65.2)	0.03
Type of neoadjuvant therapy:			-
Chemotherapy	24 (32.0)	5 (33.3)	
Radiotherapy	1 (1.3)	0	
Chemo-radiotherapy	50 (66.7)	10 (66.7)	
Hospital stay (days)^a^	19 (16–24)	23 (18–30)	0.04
Pathological stage^d^			0.07
0	23 (26.7)	2 (8.7)	
I	16 (18.6)	4 (13.0)	
II	14 (16.3)	10 (43.5)	
III	29 (33.7)	7 (30.4)	
IV	4 (4.7)	1 (4.4)	
Post-operative morbidity	20 (23.3)	9 (39.1)	0.18

Data expressed as n (%) or ^a^median (IQR)

^b^Other histotype in one patient

^c^Comparison biased due to age penalization in score calculation

^d^Pathological stage is ypstage for patients who received neoadjuvant therapy and pstage for patients who did not

### Health-related quality of life

Unadjusted raw scores of selected EORTC aspects in older and younger patients are shown in Additional file 1: Table S1 for descriptive purpose. Differences in HRQL between older and younger patients have been assessed with linear mixed-effect models and are shown as MDs in Tables 3 and 4. The effects of age group, time and group-by-time interaction from the first stage of model estimation are presented in Additional file 2: Table S2. Global quality of life and the C30 functional scales in Table 3 did not show a significant group-by-time interaction, thus indicating that the difference between older and younger patients persisted over time. In such case, the interaction factor was removed from the model and a single MD was estimated regarding the whole period. The estimated MDs were clinically not significant (MDs < 10 points, not requiring statistical comparison; Table 3) and indicated similar global quality of life (Fig. 1) and C30 functional scales over time in older and younger patients. Among symptoms, fatigue, dyspnoea and dysphagia did not show a significant group-by-time interaction, thus the interaction factor was removed from the model and a single MD was estimated regarding the whole period. Older and younger patients reported clinically similar fatigue, dyspnoea and dysphagia over time (MDs < 10 points, not requiring statistical comparison; Table 3).

**Table 3:** Health-related quality of life in older patients (age > =70) compared with younger patients (age < 70), at admission, at discharge and at 3 months after surgery, without a significant time interaction

EORTC aspect	MD (95 % C.I.)	*p*-value
C30 global quality of life	4.4 (−5.2, 14.1)	n.a.
C30 physical function	−3.1 (−10.9, 4.8)	n.a.
C30 role function	9.3 (−4.3, 23.0)	n.a.
C30 emotional function	−0.5 (−10.6, 9.6)	n.a.
C30 cognitive function	−5.9 (−13.5, 1.7)	n.a.
C30 social function	−2.3 (−14.5, 9.8)	n.a.
C30 fatigue	3.2 (−6.7, 13.1)	n.a.
C30 dyspnoea	−3.6 (−13.5, 6.3)	n.a.
OES18 dysphagia	−2.8 (−14.2, 8.6)	n.a.

Mean score difference (MDs) were assessed with linear mixed-effect models and adjusted for time

Charlson Comorbidity Index, neoadjuvant therapy, tumour stage, gender, histology, tumour site and complications. All these values did not show a significant time interaction term, i.e. the difference persisted over time. Any MD ≥ 10 was considered clinically significant and tested for statistical significance (n.a., not applicable, if MD < 10)

**Table 4:** Differences in health-related quality between older patients (age > =70) and younger patients (age < 70), at admission, at discharge and at 3 months after surgery, with significant time interaction

OES18 aspects	Admission		Discharge		3 months after surgery	
	MD (95 % C.I.)	*p*-value	MD (95 % C.I.)	*p*-value	MD (95 % C.I.)	*p*-value
Trouble swallowing saliva	17.4 (3.6, 31.2)	0.01	−7.5 (−20.7, 5.7)	n.a.	0,2 (−18.9, 19.3)	n.a.
Choking when swallowing	13.8 (5.8, 21.8)	0.0008	−0.5 (−7.9, 6.9)	n.a.	−2.5 (−13.6, 8.6)	n.a.
Eating	20.1 (7.4, 32.8)	0.002	2.4 (−9.5, 14.3)	n.a.	0.5 (−17.2, 18.2)	n.a.

Mean score difference (MDs) were assessed with linear mixed-effect models and adjusted for time

Charlson Comorbidity Index, neoadjuvant therapy, tumour stage, gender, histology, tumour site and complications. Any MD ≥ 10 was considered clinically significant and tested for statistical significance (n.a., not applicable, if MD < 10)

There was a significant group-by-time interaction for trouble swallowing saliva, choking when swallowing and eating difficulties (Table 4). These results indicated that MDs varied over time and required the estimation of MDs at each time point. Older patients reported clinically and statistically significantly worse swallowing saliva (MD = 17.4, *p* = 0.01), choking when swallowing (MD = 13.8, *p* = 0.0008) and eating difficulties (MD = 20.1, *p* = 0.002) at admission than younger patients (Fig. 1). However, MDs were not clinically significant (less than 10 points) at discharge and at 3 months after surgery, thus did not require any further statistical comparisons and were indicated as “not appliacable” in Table 4.

## Discussion

Population ageing has profound implications on social and health-care systems, since elderly have specific issues that require careful consideration. Oncology research needs to adapt accordingly, in order to identify and implement tailoring treatment for older patients [27, 28]. Several studies demonstrated that advanced age per se should not be considered a contraindication to oesophageal resection for cancer, in term of morbidity and mortality [6–9, 29]. However, HRQL impairment after oesophagectomy for cancer has been widely reported [14, 25, 30–32], thus new strategies to improve such outcome warrant future investigations. In particular, to our knowledge, no studies focused on HRQL after oesophagectomy in elderly yet. Therefore, the present study aimed to compare the effect of oesophagectomy in term of HRQL in older and younger patients.

In our series, overall quality of life was clinically similar in older and younger patients over time, as well as physical, emotional, cognitive and social self-perceptions. This finding is noteworthy because advanced age is usually associated to more comorbidities [29] and the comorbidity status has been identified as responsible for poor short term HRQL [33]. In our sample, older candidates for surgery had slightly worse comorbidity status according to CCI, whereas ACCI showed a larger difference due to age penalization in index calculator [22]. The overall frailty of older patients needs to be taken into account especially after surgery, thus our older patients had longer hospital stay than younger ones. The comorbidity status and the physiological changes associated with advanced age are often responsible for clinicians’ reluctance to have elderly patients undergo recommended treatment modalities for cancer [4, 27]. In fact, neoadjuvant therapy is usually less reported to be performed in older patients, even if some authors suggest that they can tolerate and benefit from chemoradiation therapy [16, 34].

Unexpectedly, older and younger patients reported clinically similar perception of two cancer-specific problems, such fatigue and dyspnoea, that should be more relevant in elderly. These results could be due to patient selection for surgery because all these patients have been evaluated fit for major surgery. In addition, our elderly patients might have lower expectancy in term of fatigue and dyspnoea recover than younger ones due to the so-called “response shift”, i.e. patients readjust their quality of life “thermostat” according to their current situation [14]. This phenomenon is similar to the “wellbeing paradox”: Brickman et al. [35] showed that, after an adaptation period of 1 year, paraplegic accident victims and lottery winners reported same level of overall wellbeing.

At admission, older patients reported more troubles regarding eating, choking when swallowing and swallowing saliva than younger ones, but these disparities were no longer reported at discharge and at 3 months after surgery. These results were obtained after adjusting for neoadjuvant therapy, thus taking into account its possible contribution on relieving the dysphagia problems. Despite these difficulties, our older patients had similar dysphagia and nutritional status at admission than younger ones. Swallowing difficulties increase in advanced age and can have serious health implications, including malnutrition, sarcopenia and reduced quality of life [36, 37]. Moreover, elderly can experience the qualitative and quantitative alteration of saliva (salivary hypofunction), that affects swallowing and quality of life [38]. However, these difficulties did not impair the nutritional status at admission and disappeared after surgery, thus their clinical relevance was minor.

In a previous study on HRQL in oesophageal cancer patients [14], postoperative complications and adjuvant therapy were independently associated to poor global quality of life after esophagectomy. Postoperative complications tended to negatively affect emotional and physical functions, thus leading to an impaired global quality of life. In the present study, the occurrence of postoperative complications has been included as confounder in the models, whereas adjuvant therapy has not been included due to the presence of pathological stage among confounders. In fact, the pathological stage has been included as indicator of tumour stage at admission for surgery (first time point) because the clinical stage could have been unreliable for our purpose due to the effect of neoadjuvant therapy [39]. Moreover, the inclusion of the tumour stage in the models allowed to take into account its implications (i.e. adjuvant therapy), avoiding the presence of redundant variables. Anyway, the association of adjuvant therapy and long-term HRQL has been suggested also in other cancers [40], thus requiring further focused investigations in oesophageal cancer survivors. In the present study, the interval between neoadjuvant therapy and surgery has not been included as confounder in the models because is usually standardized in our patients (6–8 weeks). In addition, it could not have been a confounder for patients who did not receive neoadjuvant therapy.

Available literature regarding the effect of age on QOL after surgery for cancer showed different results according to cancer type. A Swedish study of 355 patients undergoing oesophagectomy for cancer reported no significant association between age and HRQL at 6 months after surgery, a part from poorer emotional function in middle-aged patients than younger patients. Anyway, HRQL outcome was categorized as good or poor by collapsing the four response categories, thus limiting the variability of the outcome [41].

Similar observations were reported also by an Italian cross-sectional study of patients who undergoing potentially curative gastrectomy for cancer. HRQL was evaluated using the Karnofski scale (measuring patient autonomy in dealing with normal life after the operation) and similar HRQL at 6 months after surgery was observed in older and younger patients [42].

Different results were reported by a German prospective study of 131 patients with non-small cell lung cancer undergoing surgical resection, where the older group showed poorer HRQL at 24 months after surgery than the younger group. In fact, despite better HRQL at discharge, older patients reported poorer physical function, role function, social function and global quality of life at long term [43].

Conflicting results have been published in prostate cancer, colorectal cancer and rectal cancer. Radical prostatectomy may cause transient or permanent urinary incontinence and erectile dysfunction, but may also relief lower urinary tract symptoms. A recent review associated younger age with better general quality of life after radical prostatectomy for cancer, underlying the importance of HRQL evaluation in treatment decision making of such patients [44]. A prospective study of 1836 men undergoing prostatectomy for cancer reported better sexual function after surgery in younger men, adjusting for a set of covariates [45]. However, in a previous prospective study of 121 men undergoing prostatectomy for cancer, older patients showed better short-term physical functioning than younger patients, adjusting for a set of covariates [46].

The effect of age on HRQL is still controversial in colorectal cancer as well, with increasing age associated to lower HRQL in some studies and to higher HRQL in others [47]. Anyway, a recent multicentre prospective study reported worse global quality of life in older patients after surgical resection, with impairment of all the functions (role, physical, emotional, cognitive and social) [48].

Regarding HRQL after surgical resection for rectal cancer, two Dutch studies reported lower physical function and sexual function in older patients than younger patients [49, 50]. However, a previous German study of 253 patients with rectal cancer reported both better sexual function and worse physical function, role function and global quality of life in older patients after surgical resection [51]. These findings suggest that further studies are required on increasing age and HRQL changes after surgical resection for cancer, but the concerns regarding quality of life should not deprive patients of the opportunity for curative surgery.

The strengths of the study include the selection *a priori* of specific aspects of EORTC questionnaires and the statistical comparison limited to clinically relevant differences, to avoid the risk for multiple testing. Furthermore, the main confounding factors (comorbidity status, neoadjuvant therapy and complications) in evaluation of quality of life were included in multivariable analysis of selected EORTC aspects.

The weaknesses of the study include the number of patients who refused to fill the questionnaires and the limited number of elderly. Refusing patients - who represent a common issue of studies on quality of life [52] - were compared to participating ones, in order to evaluate possible selection bias. Refusing patients had shorter hospital stay and lower rate of neoadjuvant therapy than participating patients, thus we think that they were maybe less in contact with oncologic personnel and less willing to participate in first-person contribution to research activity. No clear information on this topic is available in literature, but two previous studies suggested that heavier users of health care might provide higher response rate [53, 54]. The tumour site was also slightly different between the two groups but, to our opinion, it did not lead to substantial bias. Such differences between refusing patients and participating ones should be considered as possible confounding factors for this study, but they could not be corrected with statistical methods. The application of adequate approaches to minimize the number of refusing patients will be warranted. Moreover, the limited number of older patients might have prevented us from identifying some differences between older and younger patients. Anyway, the shape of most confidence intervals did not suggest any substantial bias in the interpretation of the results, because most confidence intervals were centred close to the zero and had limited breadth. In addition, our series included only few patients with cervical and thoracic ESCC, because of the recent increment of EAC in Western countries. This facet needs to be taken into account by readers from Eastern countries, where ESCC still remains the more common EC hystotype [55].

## Conclusions

Early HRQL perception after surgery of older patients resulted comparable to that of their younger counterparts. This result may also be due to some predisposition of the elderly to adapt to the new status. Studies on pain control and minimally invasive procedure will be warranted.

## Additional files

Additional file 1: Table S1. Unadjusted raw scores of selected EORTC aspects in older and younger patients. (DOC 49 kb)
Additional file 2: Table S2. Effect of age group, time and group-by-time interaction from the first stage of the estimation of linear mixed-effect models (adjusted for Charlson Comorbidity Index, neoadjuvant therapy, tumour stage, gender, histology, tumour site and complications). (DOC 44 kb)

## Abbreviations

ACCI: Age related Charlson comorbidity index
CCI: Charlson comorbidity index
HRQL: Health-related quality of life
MD: Mean score difference
PNI: Prognostic nutritional index
