# Supplementary material for: Health related quality of life after oesophagectomy: elderly patients refer similar eating and swallowing difficulties than younger patients
Source: BMC Cancer. 2015 Sep 21;15:640. doi: 10.1186/s12885-015-1647-5 (PMC4578681; doi:10.1186/s12885-015-1647-5)
Supplement: Additional file 1: Table S1. — Unadjusted raw scores of selected EORTC aspects in older and younger patients. (DOC 49 kb) [file 12885_2015_1647_MOESM1_ESM.doc]

Supplementary Table S1. Unadjusted raw scores of selected EORTC aspects in older and younger patients.

|  | Age<70 | | | Age>=70 | | |
| --- | --- | --- | --- | --- | --- | --- |
| Time | Admission | Discharge | 3 months after surgery | Admission | Discharge | 3 months after surgery |
| C30 QL | 68.4 (21.7) | 54.2 (19.0) | 67.5 (15.9) | 61.1 (27.0) | 64.5 (23.2) | 72.3 (19.6) |
| C30 PF | 86.6 (18.4) | 71.1 (20.9) | 76.3 (34.1) | 86.1 (12.0) | 65.9 (20.8) | 74.9 (26.4) |
| C30 RF | 81.9 (26.9) | 51.2 (34.9) | 73.1 (28.6) | 90.4 (18.2) | 64.0 (33.0) | 79.2 (22.5) |
| C30 EF | 75.0 (21.0) | 74.3 (23.8) | 77.9 (20.5) | 75.4 (22.0) | 75.0 (25.3) | 79.1 (31.3) |
| C30 CF | 89.9 (17.5) | 83.3 (17.1) | 87.5 (26.4) | 87.1 (13.8) | 77.4 (18.9) | 84.7 (21.9) |
| C30 SF | 81.2 (24.5) | 70.8 (30.4) | 77.2 (23.6) | 87.2 (20.6) | 73.3 (28.3) | 80.6 (30.8) |
| C30 FA | 23.2 (21.7) | 43.1 (24.6) | 31.1 (23.8) | 21.4 (23.8) | 45.6 (21.4) | 30.9 (31.8) |
| C30 DY | 10.3 (19.9) | 26.4 (24.8) | 15.3 (19.1) | 14.2 (21.5) | 23.3 (21.9) | 22.2 (29.6) |
| OES18 DYS | 62.5 (34.0) | 49.8 (23.2) | 49.4 (21.9) | 61.6 (29.1) | 49.1 (30.1) | 44.5 (34.9) |
| OES18 SSV | 4.2 (17.7) | 8.3 (17.4) | 3.8 (12.6) | 17.8 (35.3) | 8.8 (15.0) | 5.5 (12.9) |
| OES18 CH | 1.9 (9.7) | 0.5 (3.9) | 1.9 (7.7) | 13.4 (30.4) | 3.3 (10.2) | 2.8 (9.6) |
| OES18 EAT | 15.6 (22.0) | 18.8 (16.4) | 20.0 (16.6) | 29.3 (28.7) | 20.9 (14.7) | 25.0 (19.5) |

Data expressed as mean(SD).

C30 aspects: QL global quality of life, PF physical function, RF role function, EF emotional function, CF cognitive function, SF social function, FA fatigue, DY dyspnoea.

OES18 aspects: DYS dysphagia, SSV trouble swallowing saliva, CH choking when swallowing, EAT eating.
