# Supplementary material for: Health related quality of life after oesophagectomy: elderly patients refer similar eating and swallowing difficulties than younger patients
Source: BMC Cancer. 2015 Sep 21;15:640. doi: 10.1186/s12885-015-1647-5 (PMC4578681; doi:10.1186/s12885-015-1647-5)
Supplement: Additional file 2: Table S2. — Effect of age group, time and group-by-time interaction from the first stage of the estimation of linear mixed-effect models (adjusted for Charlson Comorbidity Index, neoadjuvant therapy, tumour stage, gender, histology, tumour site and complications). (DOC 44 kb) [file 12885_2015_1647_MOESM2_ESM.doc]

Supplementary Table S2. Effect of age group, time and group-by-time interaction from the first stage of the estimation of linear mixed-effect models (adjusted for Charlson Comorbidity Index, neoadjuvant therapy, tumour stage, gender, histology, tumour site and complications).

| HRQL aspect | Age group  (p-value) | Time  (p-value) | Group-by-time interaction  (p-value) |
| --- | --- | --- | --- |
| C30 global quality of life | 0.34 | 0.57 | 0.10 |
| C30 physical function | 0.31 | 0.0006 | 0.68 |
| C30 role function | 0.24 | 0.0008 | 0.86 |
| C30 emotional function | 0.83 | 0.85 | 0.93 |
| C30 cognitive function | 0.18 | 0.10 | 0.76 |
| C30 social function | 0.87 | 0.06 | 0.73 |
| C30 fatigue | 0.46 | <0.0001 | 0.53 |
| C30 dyspnoea | 0.76 | 0.04 | 0.52 |
| OES18 dysphagia | 0.75 | 0.0002 | 0.82 |
| OES18 trouble swallowing saliva | 0.32 | 0.02 | 0.0005 |
| OES18 choking when swallowing | 0.20 | 0.008 | 0.009 |
| OES18 eating | 0.09 | 0.58 | 0.009 |

A statistically significant interaction term (p<0.01) suggested that the difference between older and younger patients were not the same for all time points; in such case, the two age groups were compared at each time point. Otherwise, a statistically not significant interaction term (p>0.01) suggested that there was no difference over time between the two age groups; in this case the interaction term was removed from the final model.
